# Recurrent Pleural Effusions Secondary to Pancreaticopleural Fistula: A Case Presentation

**DOI:** 10.7759/cureus.41625

**Published:** 2023-07-10

**Authors:** Ali Tariq Shaikh, Ibrar Atiq, Saqib Gul, Owais Gul, Wajiha Ali

**Affiliations:** 1 Internal Medicine, United Health Services Wilson Medical Center, Johnson City, USA; 2 Internal Medicine, Hamdard College of Medicine & Dentistry, Karachi, PAK

**Keywords:** pancreaticopleural fistula, pancreatic pseudocysts, chronic pancreatitis, ercp, pleural effusion

## Abstract

Pleural effusion can be a complication of pancreatic diseases. Pancreaticopleural fistula (PPF) is a rare complication arising as a result of chronic pancreatitis that causes recurrent pleural effusions often resistant to thoracentesis. Diagnosis of PPF can be delayed, and presentation with respiratory symptoms related to pleural effusion is common. Elevated pleural fluid amylase and lipase levels are always helpful, but final diagnosis mostly requires demonstration of fistula on imaging modalities, such as computed tomography (CT) scan or magnetic resonance cholangiopancreatography (MRCP). Endoscopic retrograde cholangiopancreatography (ERCP) serves as a diagnostic and therapeutic tool. Here, we present a case of PPF leading to recurrent pleural effusions, treated with stent placement.

## Introduction

The correlation between pancreatic diseases and pleural effusions is widely acknowledged [1]. Acute pancreatitis often presents with small, temporary effusions that are typically located on the left side. These effusions are believed to be caused by either lymphatic or sympathetic factors. By contrast, chronic pancreatic disease, with or without the presence of pseudocysts, can be associated with recurring pleural effusions of a larger size [1,2]. 

Pancreaticopleural fistula (PPF) is a rare complication that can arise from chronic pancreatitis and has only been acknowledged in the past few decades. It occurs with a disruption in the main pancreatic duct leading to the formation of a fistula between the pancreas and the pleural space or by extension of a pancreatic pseudocyst into the mediastinum. This causes pancreatic fluid to flow through the retroperitoneum and reach the thoracic cavities [3]. Typically, patients exhibit symptoms related to the pleural effusion, while the underlying pancreatic disease often remains symptomless. Due to the rarity of the condition and the challenges in radiologically detecting the fistula, the diagnosis is often delayed [3,4]. 

Here, we report a patient who presented with large recurring pleural effusions due to a pancreatic-pleural fistula and underwent diagnostic and therapeutic endoscopic retrograde cholangiopancreatography (ERCP).

## Case presentation

A 63-year-old male with a past medical history of chronic alcoholic pancreatitis complicated with pancreatic pseudocysts, chronic hyponatremia, benign essential hypertension, and rheumatoid arthritis presented to the hospital with chest pain, shortness of breath, and cough for the past five days. Previously, he was evaluated at another facility for recurrent pancreatitis episodes; he had at least three episodes during last two years, and the last episode was only two months ago. He described his chest pain as sharp, non-radiating, localized in the right rib cage, with maximum 6/10 in intensity, and worsened with deep breathing. He was feeling short of breath on exertion and on rest and denied any orthopnea or paroxysmal nocturnal dyspnea (PND). His cough was productive with white-colored sputum. The review of systems was positive for fatigue and loss of appetite but negative for any fever, chills, weight loss, nausea, vomiting, diarrhea, or abdominal pain. 

On physical exam, he was noted to be tachycardiac and tachypneic; his heart rate was 110 per minute, respiratory rate was 24 breaths per minute, blood pressure was 113/75 mmHg, and temperature was 97.1°F. He was noted to be hypoxic; oxygen saturation was 83% on room air, so he had to be put on 3 L oxygen via nasal cannula. The patient was alert and oriented. Breath sounds were decreased on the right side of the chest, and there was dullness to percussion on the affected side. Heart sounds were regular with normal S1 and S2. His abdomen was soft, non-distended, and non-tender with normal bowel sounds.

**Figure 1:**
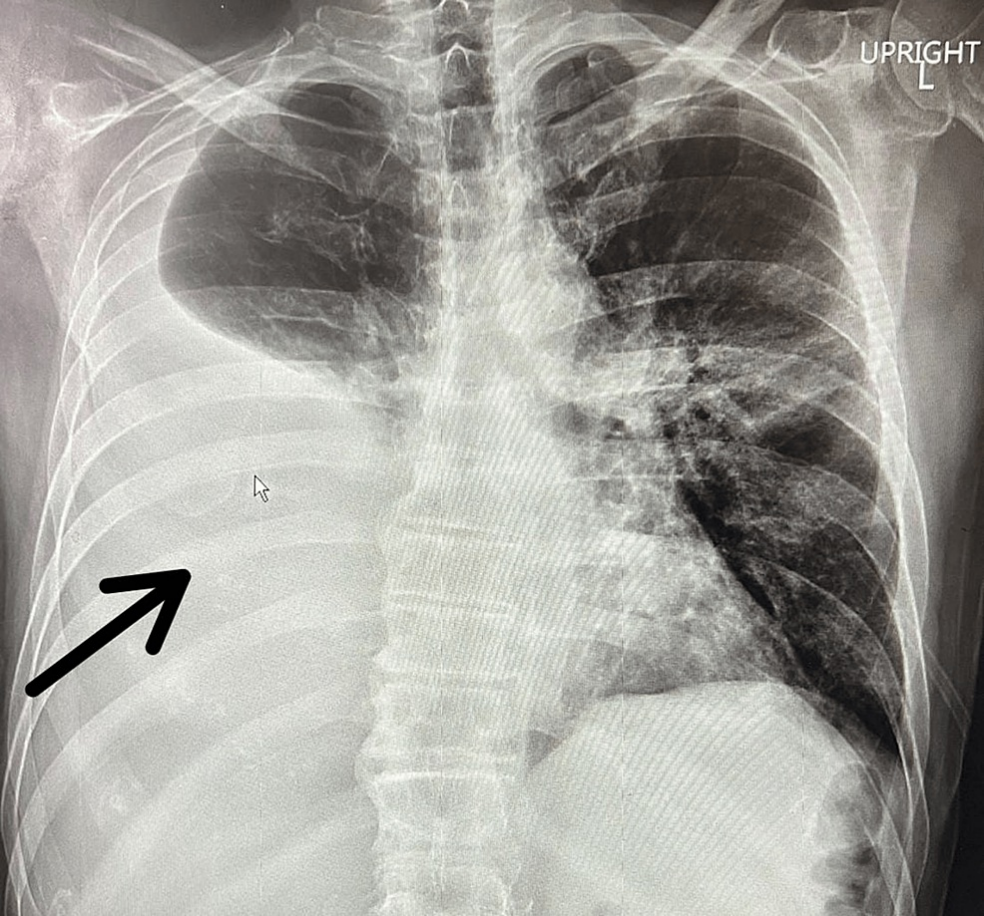
Chest X-ray showing a large right-sided pleural effusion.

**Figure 2:**
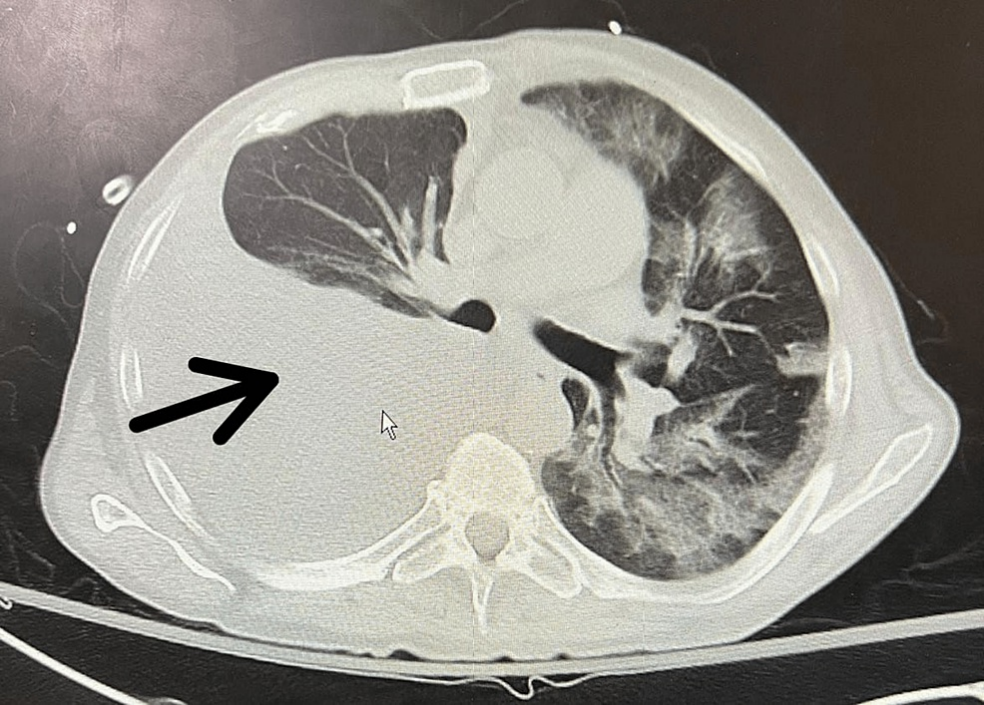
Chest CT scan without contrast showing a large right-sided pleural effusion leading to complete collapse of right middle and lower lobes. CT: computed tomography

**Figure 3:**
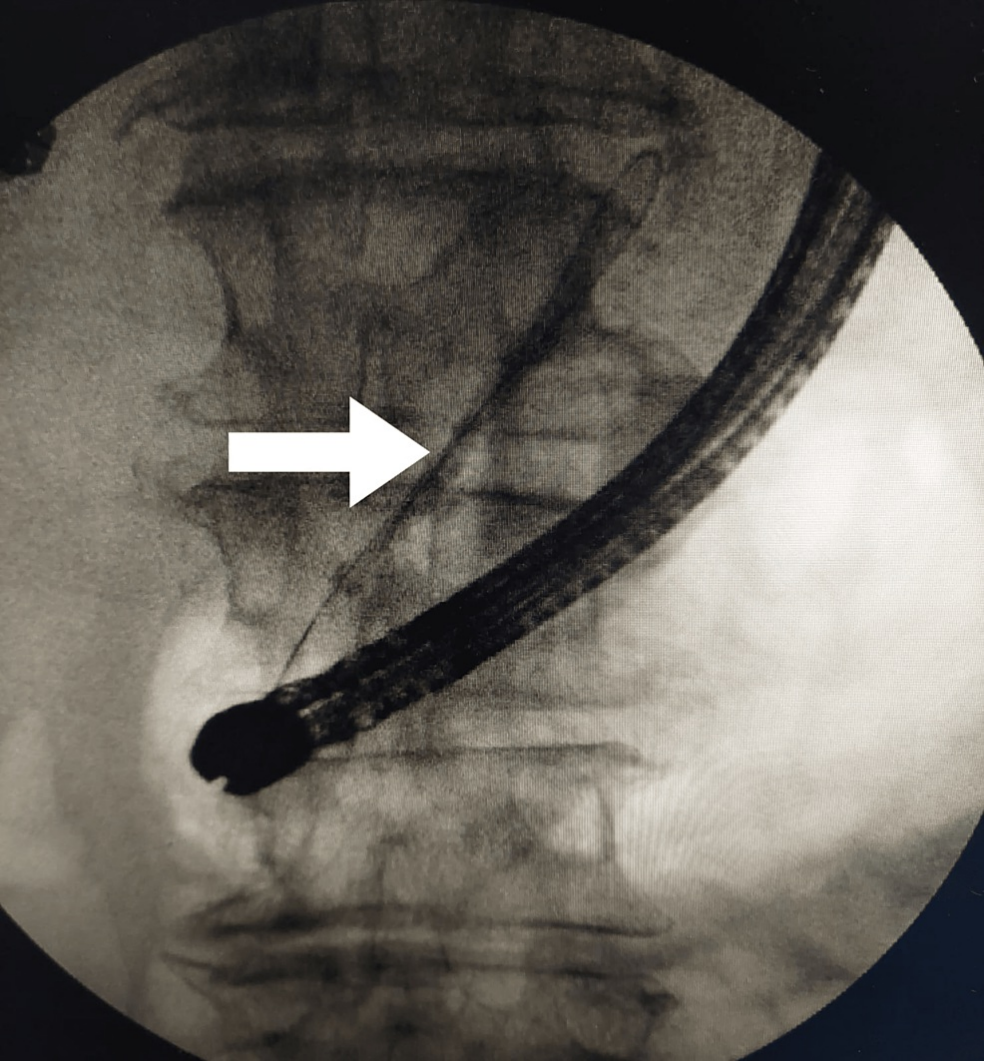
Endoscopic retrograde cholangiopancreatography showing stent being advanced into the ventral pancreatic duct.

Laboratory workup showed elevated white blood cell count of 11.5 x 10E9/L, decreased serum sodium of 130 mEq/L, elevated serum amylase of 496 U/L, elevated serum lipase of 1186 U/L, elevated C-reactive protein of 6.8 mg/dL, and elevated procalcitonin levels of 1.1 ng/mL. Chest X-ray revealed a large right-sided pleural effusion with complete collapse of the right middle and lower lobes (Figure 1). Computed tomography (CT) scan of the chest, abdomen, and pelvis without contrast was obtained that confirmed a large right-sided pleural effusion pushing mediastinum toward the left side (Figure 2). Coarse calcifications were seen throughout the pancreas representing chronic pancreatitis. Moreover, there were multiple cysts noted in the pancreas, the largest one measuring 2.9 cm in the pancreatic head, likely representing pancreatic pseudocysts. The patient was started on intravenous (IV) antibiotics for the empiric treatment of community-acquired pneumonia. He underwent two back-to-back diagnostic and therapeutic thoracentesis on alternative days followed by an indwelling pleural catheter placement that caused a significant drainage of more than 2 L. The results of pleural fluid analysis showed pH of 7.36, leukocyte count of 293 cells/mm3, triglycerides of 9 mg/dL, total protein of 3.7 g/dL, and lactate dehydrogenase (LDH) of 682 U/L. Gram stain and culture studies were negative. Serum total protein was noted to be 6.7 g/dL, and serum LDH was 258 U/L. As per Light’s criteria, pleural fluid was found to be exudative in nature. Further testing was done to rule out relatively rare causes, such as the pancreatic origin of pleural fluid. Pleural fluid amylase and lipase were found to be profoundly elevated, i.e., 42920 and 24320 U/L, respectively. The patient was then transferred to the higher-level-of-care center for the possible need of PPF repair. At that facility, he underwent a CT chest and abdomen with contrast that showed fluid collection adjacent to the pancreas with a tract extending through the right diaphragmatic crus to the right pleural space. The patient then underwent ERCP that revealed a significant leak in the pancreatic duct between the body and tail of the pancreas. A plastic stent was placed into the ventral pancreatic duct (Figure 3), and the patient was discharged with a six-week follow-up advised for pleural catheter removal. During those six weeks, the patient had serial chest X-rays done that showed interval improvement in pleural effusion. Pleural fluid amylase levels were also repeatedly checked, and they were noted to be down-trending to 49 U/L. The patient’s output from the pleural catheter decreased to less than 50 mL/day, after which it was successfully taken out. The patient underwent repeat ERCP three months later that revealed successful healing of the fistulous tract, so the stent was removed and he was discharged home. 

## Discussion

PPFs are a rare occurrence that can arise as a complication of chronic pancreatitis. The frequency reported in the literature ranges from 0.4% to 4.5% among individuals diagnosed with pancreatitis [1,2]. It arises when pancreatic fluid enters the pleural cavity due to a rupture of the pancreatic duct caused by a trauma or surgery [5]. An alternative cause can be the presence of a pancreatic pseudocyst that forms a fistula connecting it to the pleural cavity [6]. The associated pleural effusion is characterized by its significant size, recurrent nature, and resistance to repeated thoracentesis. Typically, it affects one side of the chest, with a left-sided predominance and infrequent occurrence on the right side (approximately 20% of cases). Bilateral involvement is uncommon, occurring in only around 15% of cases [5,6].

The clinical presentation frequently leads to confusion and often results in a delayed diagnosis. In Rockey and Cello's analysis, the typical duration of symptoms was found to be 5.6 weeks [3]. Patients with PPFs experience cardiovascular and respiratory symptoms more frequently than gastrointestinal symptoms [6]. Our patient presented similarly with respiratory symptoms, such as dyspnea, cough, and chest pain, while no abdominal symptoms were reported.

In a patient who presents with a new pleural effusion on the left side and has a history of pancreatitis and/or heavy alcohol abuse, the diagnosis of a PPF should be taken into consideration. This can be determined by observing the findings of an exudative fluid sample obtained through thoracentesis, which exhibits significantly elevated levels of amylase and lipase [7]. Our patient had a similar presentation with pleural fluid analysis depicting an exudative effusion with an amylase level of 42,920 IU/L and a lipase level of 24,320 IU/L. These findings align with the pleural amylase levels reported in the review conducted by Rockey and Cello, where the mean recorded value was 18,450 IU/L (range: 1,830-164,187 IU/L) [3].

Once the suspicion of a PPF is confirmed, there are multiple imaging modalities that can be utilized to visualize the fistula. Currently, the most commonly employed modalities in clinical practice are CT, ERCP, and magnetic resonance cholangiopancreatography (MRCP) with 47%, 78%, and 80% sensitivity, respectively [8].

In our patient, a CT scan without contrast showed chronic pancreatic calcifications and the presence of multiple pseudocysts. Later, CT with contrast study hinted toward the presence of a fistulous tract. Eventually, the patient underwent ERCP, which served as a diagnostic and therapeutic approach. While CT scans of the thorax and abdomen have been successful in identifying PPF, their ability to accurately delineate the fistula remains a subject of debate, and their sensitivity is comparatively lower than that of the other imaging modalities. Nevertheless, CT remains valuable in demonstrating certain pancreatic abnormalities, such as parenchymal atrophy, calcification, duct dilatation, and pseudocysts [9].

ERCP offers the advantage of directly visualizing the papilla and the surrounding anatomy. It also enables simultaneous therapeutic procedures during the endoscopic examination. However, it has its limitations. ERCP is noted to be an invasive procedure and may not clearly visualize a fistula if the site of ductal disruption is distal to an obstruction, such as in the presence of a stricture [8]. Moreover, the accuracy of ERCP can vary significantly and depends on the skills of the operator, the timing of the procedure, and the presence of anatomical variations. Due to its limitations and the potential for severe complications, such as infection, pancreatitis, bleeding, and perforation, ERCP is not recommended as the primary diagnostic tool for confirmation. At present, MRCP has emerged as the preferred imaging modality for diagnosing PPFs. This preference stems from its noninvasive nature and its ability to visualize the fistula even in the presence of strictures. Despite its limited therapeutic capabilities, MRCP is considered an imaging method of choice for PPFs due to these reasons. Precise visualization of the site and its anatomical relationship with the pancreatic ductal tree is especially beneficial when planning surgical intervention or if surgical treatment is being considered [10].

Conservative measures have shown success in healing up to 50% of internal pancreatic fistulas and 70% to 90% of external pancreatic fistulas [11]. In cases where patients have a dilated main pancreatic duct without disruption or stricture, conservative therapy is recommended. This approach involves the use of broad-spectrum antibiotics, enteral nutrition, and addressing fluid and electrolyte imbalances [12]. In addition, the administration of somatostatin analogs has been reported to facilitate the closure of pancreatic fistulas by reducing the output volume of the fistulous tract [13].

When conservative measures fail or complications, such as infection or bleeding arise, endoscopic or surgical interventions are necessary for managing pancreatic fistulas [14]. ERCP is a safe and reliable modality that can be regarded as the primary treatment option. Early ERCP with pancreatic stent insertion can help resolve the fistula and potentially prevent the need for surgery. Surgical interventions, with their associated risks, should be reserved for cases unresponsive to conservative measures [15]. In this case, the patient underwent repeated thoracentesis with eventual ERCP and stent placement into the ventral pancreatic duct. An indwelling catheter was kept in place while the fistulous track was allowed to heal on its own.

## Conclusions

PPF is an exceedingly rare manifestation in patients with a history of pancreatitis. Affected patients can present with respiratory symptoms pertaining to recurrent pleural effusions. Various imaging methods, such as ERCP, MRCP, and CT scan, can aid in diagnoses. Initially, non-operative approaches, including medical and endoscopic interventions, can be attempted for managing a PPF. If conservative methods prove ineffective, surgical options may be considered.
